# Transcriptional Responses of Different Brain Cell Types to Oxygen Decline

**DOI:** 10.3390/brainsci14040341

**Published:** 2024-03-30

**Authors:** Camille Ravel-Godreuil, Ethan R. Roy, Srinivas N. Puttapaka, Sanming Li, Yanyu Wang, Xiaoyi Yuan, Holger K. Eltzschig, Wei Cao

**Affiliations:** 1 Department of Anesthesiology, Critical Care and Pain Medicine, McGovern Medical School, University of Texas Health Science Center at Houston, Houston, TX 77030, USA; camille.m.godreuil@uth.tmc.edu (C.R.-G.); ethan.r.roy@uth.tmc.edu (E.R.R.); sputtapa@bidmc.harvard.edu (S.N.P.); sanming.li@uth.tmc.edu (S.L.); yanyu.wang@uth.tmc.edu (Y.W.); xiaoyi.yuan@uth.tmc.edu (X.Y.); holger.eltzschig@uth.tmc.edu (H.K.E.); 2 Division of Gastroenterology, Department of Medicine, Beth Israel Deaconess Medical Center, Harvard Medical School, Boston, MA 02115, USA

**Keywords:** hypoxia, neuron, astrocyte, microglia, hypoxia-inducible factors, cerebral hypoxia, hypoxia gene module, oxygen deprivation

## Abstract

Brain hypoxia is associated with a wide range of physiological and clinical conditions. Although oxygen is an essential constituent of maintaining brain functions, our understanding of how specific brain cell types globally respond and adapt to decreasing oxygen conditions is incomplete. In this study, we exposed mouse primary neurons, astrocytes, and microglia to normoxia and two hypoxic conditions and obtained genome-wide transcriptional profiles of the treated cells. Analysis of differentially expressed genes under conditions of reduced oxygen revealed a canonical hypoxic response shared among different brain cell types. In addition, we observed a higher sensitivity of neurons to oxygen decline, and dissected cell type-specific biological processes affected by hypoxia. Importantly, this study establishes novel gene modules associated with brain cells responding to oxygen deprivation and reveals a state of profound stress incurred by hypoxia.

## 1. Introduction

For almost all life forms on earth, oxygen is an essential nutrient for tissue oxidation and energy metabolism. When oxygen levels decline, mammals elicit a rapid adaptive reaction termed hypoxia response, which markedly alters the whole-body physiology to ensure survival [1]. At the individual level, immediately, the heart rate increases, and the blood vessels dilate to boost oxygen delivery to the stressed tissues. Meanwhile, at the cellular level, a substantial transcriptional program is instigated to express genes that encode proteins needed in adaptation to low oxygen availability, such as increasing red blood cell production and altering metabolic pathways to generate energy [1,2,3,4,5,6,7,8,9].

Hypoxia-inducible factors (HIF) are the master transcriptional regulators of the hypoxia response [2,3,6,7]. Oxygen-labile HIF alpha subunits are stabilized under low oxygen conditions and, in association with HIF beta subunits, control the induction of various genes encoding proteins related to angiogenesis, erythropoiesis, and anaerobic metabolism [10,11]. Three HIFα protein isoforms exhibit highly conserved domain structure and bind to the same hypoxia-responsive element (HRE) DNA motif to drive gene expression [12,13]. Under normoxia conditions, proline residues of the HIFα subunits are hydroxylated by prolyl hydroxylase domain (PHD) proteins in an oxygen-dependent manner [14]. Subsequently, the hydroxylated HIFαs are recognized and targeted by von Hippel–Lindau (VHL) tumor suppressor protein for ubiquitylation and proteasomal degradation [15]. When oxygen is limited, non-hydroxylated HIFαs translocate to the nucleus to initiate the transcription of a large panel of hypoxia-responsive genes.

The brain is the body’s most energy-demanding organ due to its high metabolic rate and relies critically on oxygen as the primary element for optimal functioning; hence, it is the organ most sensitive to oxygen deprivation [16]. Cerebral hypoxia is an emergent medical condition where the brain cannot obtain sufficient oxygen and can occur as a result of suffocation, cardiac arrest, drowning, high altitude exposure, respiratory rest, and stroke. Patients with brain hypoxia may manifest mild symptoms, including memory loss and difficulties with motor function, but in severe cases, cerebral hypoxia can result in coma, seizures, or brain death.

The response within a tissue to hypoxia may vary significantly between different cell types, depending on the metabolic demands and functional specialization of the cells. The brain is one of the most complex organs and consists of diverse types of cells that form intricate networks and connections with one another [17]. In the nervous system, neurons are the fundamental cells that transmit and receive electrical signals to maintain bodily functions and enable brain activities [18]. Astrocytes, named after their star-shaped bodies, are a major glial cell population that provides essential support and protection to neurons in various ways [19]. Microglia, on the other hand, are the brain-resident immune cells and play crucial roles in defense response, debris removal, and developmental support [20]. Previous investigations have revealed that neurons respond to hypoxia with significant gene expression alteration and metabolic reprogramming [21,22,23], but whether astrocytes and microglia react similarly is unknown. Depending on the severity of oxygen deprivation, hypoxia response can have adaptive effects or lead to pathogenic outcomes [5]. It remains unclear how a mild hypoxia response in brain cells differs from a strong one at the transcriptome level.

In this study, we exposed mouse primary neurons, astrocytes, and microglia cells to normoxia and two different hypoxia conditions and examined the transcriptomes of the treated cells. By analyzing differentially expressed genes and enriched functional pathways, we detected a conserved hypoxia response among the brain cells and observed cell type-specific sensitivity and transcriptional responses to oxygen decline. Importantly, our study, for the first time, established novel gene modules associated with brain cells responding to different extents of oxygen deprivation and revealed a state of overwhelming stress induced by hypoxia on brain cells.

## 2. Methods

### 2.1. Animals

C57BL/6J mice bred from a line originally obtained from the Jackson Laboratory were used in this study. Mice were housed in groups of 2–5 per cage under conventional housing conditions and a standard light/dark cycle. Both male and female neonates were used in experiments.

### 2.2. Primary Mouse Brain Cell Cultures

The methods used for culturing primary neurons and microglia were described previously [24,25]. To culture neurons, forebrain hemispheres from P0 pups were dissected and the dissociated cells were cultured in completed neuronal culture media (Neurobasal medium supplemented with 2% B27, 0.5 mM L-glutamine, 40 U/mL penicillin, and 40 μg/mL streptomycin). Cells were maintained in incubators at 37 °C, 5% CO_2_, and half the culture medium was replaced every 5–7 days. To culture astrocytes, P1–P3 mouse brains were dissected under a dissecting microscope in ice-cold dissection buffer (1x HBSS supplemented with 10 mM HEPES, pH 7.5, 0.6% glucose, 1% Pen-Strep). The cortex, together with the hippocampus, was digested in trypsin (2.5%) at 37 °C for 15 min, followed by the addition of soybean trypsin inhibitor (1 mg/mL) and DNase I (1%). Dissociated brain cells were resuspended and cultured with AstroMACS medium (Miltenyi Biotec, #130-117-031, Auburn, CA, USA) 10–14 days before use. Mouse microglia cells from mixed glial cell culture were selected with CD11b microbeads according to the manufacturer’s instruction (#130-093-634, Miltenyi Biotec). Cells were cultured in glial culture medium (DMEM, 10% FBS, 1% (*v*/*v*) Pen-Strep) supplemented with 10% (*v*/*v*) of conditional medium harvested from confluent LADMAC cell cultures (ATCC^®^ CRL-2420™) and 10 ng/mL of rTGFβ1 (Tonbo Bioscience, San Diego, CA, USA).

### 2.3. Hypoxia Treatments

Primary brain cells were cultured in 24 well plates in replicates under normoxia until mature. Prior to treatment, half of the culture medium in which primary neurons and astrocytes matured under normoxic conditions was replaced with a warm medium pre-equilibrated to the corresponding level of O_2_ per condition. For microglia, complete medium change was performed with pre-equilibrated medium. Cellplates were then left inside either a Whitley H35 Hypoxystation (Don Whitley Scientific Limited, Shipley, United Kingdom) set at 1% or 5% O_2_ levels, or a Cell IQ^TM^ inCusaFe CO_2_ incubator (PHCbi, PHC Corporation, Minato City, Tokyo, Japan) in normal atmosphere. Cells were incubated for 8 h and lysed immediately with TRIzol solution inside the hypoxic chamber or a regular tissue culture hood.

### 2.4. Library Preparation and Transcriptome Sequencing

Total RNA was extracted with Direct-zol RNA Microprep Kit (Cat# R2062, ZYMO Research). Novogene Co. (Sacramento, CA, USA) performed mRNA sequencing and data analysis. Basically, the RNA quality was evaluated as follows: RNA integrity number > 7.0 and 28S:18S ratio > 1.8. Messenger RNA was purified from total RNA using poly-T oligo-attached magnetic beads for library construction. The library cDNA was subjected to paired-end sequencing with a pair-end 125-base pair reading length on an Illumina HiSeq 2500 sequencer (Illumina, San Diego, CA, USA).

### 2.5. RNA-Seq Analysis

For quantification of gene expression, featureCounts (v1.5.0-p3) was used to calculate the read numbers mapped to each gene. Subsequently, the FPKM of each gene was determined based on the length of the gene and read counts mapped to the gene. Differential expression analysis was performed using the DESeq2 R package (1.20.0). The *p*-values were adjusted (*P*_adj_) using Benjamini and Hochberg’s approach for controlling the false discovery rate. For all analyses in this study, only genes with fold change > 1.2 and *P*_adj_ < 0.05 were considered differentially expressed.

### 2.6. Principal Component Analysis

Gene expression counts data for all samples was uploaded to ExpressAnalyst (https://www.expressanalyst.ca/; accessed on 26 January 2024) for filtering (unannotated features, count abundance < 4, and variance percentile rank < 15) and normalization (log2-counts per million transformation). Filtered and normalized data was then uploaded into ClustVis (https://biit.cs.ut.ee/clustvis/; accessed on 26 January 2024) to perform principal component analysis, and PC values were plotted in GraphPad Prism. Ellipses were drawn manually in plots to encompass groups.

### 2.7. Pathway Analysis

For all pathway analyses presented in the figures, genes up- or downregulated (fold change > 1.2, *P*_adj_ < 0.05) were queried using the KEGG database in g:Profiler [26] (https://biit.cs.ut.ee/gprofiler/gost; accessed on 26 January 2024) with significance threshold of 0.05 (based on the g:SCS algorithm for multiple testing correction) and plotted using Prism.

### 2.8. Statistics

All data in bar plots are presented as −log_10_(*P*_adj_) of KEGG pathways (or numbers of genes as in Figure S1). Violin plots with individual sample values, medians, and quartiles were used to plot the expression (FPKM) of individual transcripts. Heatmaps were generated using group-averaged FPKM values. Shapiro-Wilk normality tests were used to assess data distribution. Differences between cell types under normoxia were analyzed by one-way ANOVA with Tukey’s post-hoc multiple-comparisons tests (or by Kruskal-Wallis test with Dunn’s post-hoc multiple comparisons test when the distribution was not normal). Differences between O_2_ levels within each cell type were analyzed by one-way ANOVA with Dunnett’s post hoc multiple-comparisons tests (or by Kruskal-Wallis test with Dunn’s post hoc multiple-comparison test when the distribution was not normal). GraphPad Prism (v10.0.0) was used for statistical analyses and plotting. *p*-values less than 0.05 were considered significant (noted as * *p*  <  0.05, ** *p*  <  0.01, *** *p*  <  0.001, **** *p*  <  0.001 in plots), and those over 0.05 were considered non-significant (“ns”). Microscopy images are representative of multiple replicates. No statistical methods were used to predetermine sample sizes. Biorender.com was used to generate all schematics within the figures.

## 3. Results

### 3.1. Exposing Primary Mouse Brain Cells to Different Oxygen Levels

**Figure 1:**
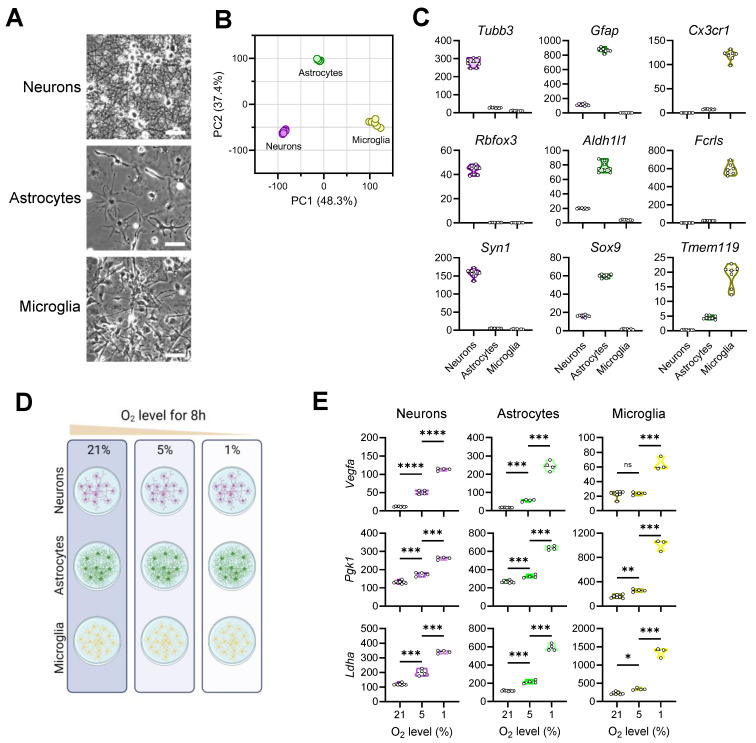
Culture of primary mouse brain cells and exposure to different levels of oxygen. (**A**): Images of mature CNS cells in culture before O_2_ treatment (scale bar = 50 µm); (**B**): PCA plots of brain cell transcriptomes at 21% O_2_; (**C**): Level of expression of brain cell makers at 21% O_2_; (**D**): Schematic of experimental plan, mature cultures of each cell type (neuron, astrocytes and microglia) were exposed to either 21, 5 or 1% of oxygen for 8 h before the RNA was extracted for sequencing; (**E**): level of expression of markers of hypoxia in each CNS cell type upon treatments. (****: *p* < 0.0001, ***: *p* < 0.001, **: *p* < 0.01, *: *p* < 0.05).

To capture brain cells’ response to oxygen variation, we first established primary cultures of three mouse brain cell types under normoxic conditions (21% O_2_). These cells each displayed characteristic morphologies and possessed distinctive transcriptomes (Figure 1A,B). As expected, neurons selectively expressed genes that encode characteristic neuronal markers such as β tubulin (*Tubb3*), NeuN (*Rbfox3*), and synapsin I (*Syn1*) (Figure 1C). Likewise, well-known astrocytic markers *Gfap*, *Aldh11*, and *Sox9* were highly elevated in astrocytes, while microglia were enriched in myeloid markers *Cx3cr1*, *Fcrls*, and *Tmem119*, validating the identities of the cultured brain cells.

We experimentally exposed the three cell types to different oxygen concentrations—1% (widely used model of hypoxia) [27,28,29], 5% (milder degree of hypoxia), and 21% (normoxia)—for 8 h in culture and performed RNAseq analysis on the resulting samples (Figure 1D). In peripheral cells, hypoxia potently induces vascular endothelial growth factor (*Vegf*) expression to stimulate angiogenesis and upregulate phosphoglycerate kinase 1 (*Pgk1*) and lactate dehydrogenase A (*Ldha*) to promote glycolysis [30,31,32]. In all three brain cell types, we observed an overall dose-dependent upregulation of these hypoxia-responsive genes (Figure 1E). Thus, we validated the experimental manipulations by detecting expected molecular responses by brain cells to oxygen decline.

### 3.2. Transcriptional Responses of Neurons to Reduced Oxygen Availability

**Figure 2:**
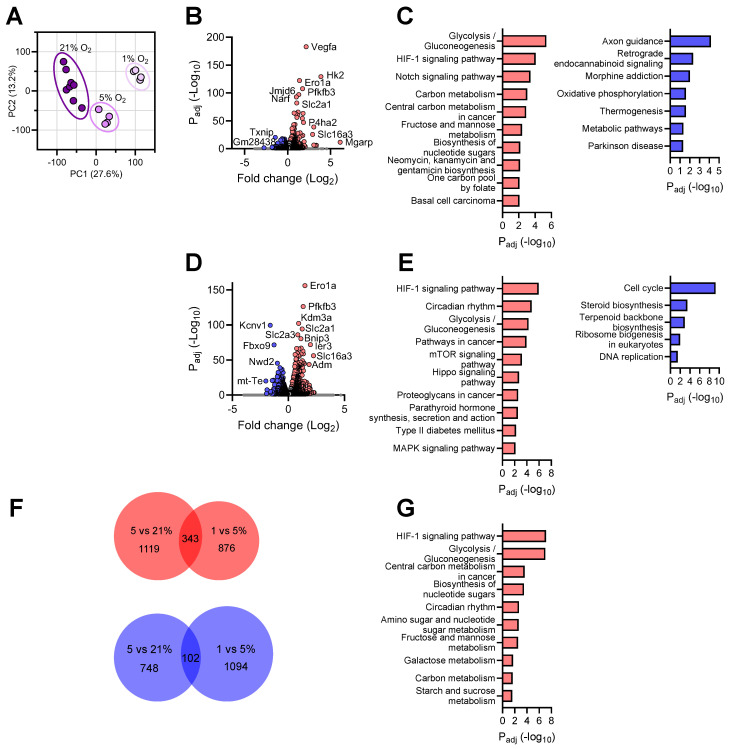
Transcriptional profile of neurons in response to different oxygen levels. (**A**): PCA plot of the transcriptomes of neuronal cultures under different O_2_ conditions; (**B**)**:** Volcano plot of significantly up- and downregulated genes at 5% vs. 21% O_2_ (FC > 1.2); (**C**): Top KEGG pathways enriched in up- (left) and downregulated (right) genes at 5% vs. 21% O_2_; (**D**): Volcano plot of significantly up- and downregulated genes at 1% vs. 5% O_2_ (FC > 1.2); (**E**): Top KEGG pathways enriched in up- (left) and downregulated (right) genes at 1% vs. 5% O_2_; (**F**): Venn diagrams showing the numbers of unique and shared DEGs (upregulated in red, downregulated in blue) between the two hypoxic conditions tested; (**G**): Top KEGG pathways enriched in upregulated genes shared between the two hypoxic conditions tested.

To comprehend the transcriptomic differences in neurons under different O_2_ conditions, we first generated a principal component analysis (PCA) plot and observed distinctly segregated clustering of the treatment groups, indicating robust global responses by neurons to oxygen decline (Figure 2A). By comparing the transcriptomes of neurons cultured under 5% O_2_ vs. those under normoxia, we detected 1462 genes significantly upregulated (Figure 2B and Figure S1). Pathway analysis of these differentially expressed genes (DEGs) identified “Glycolysis/Gluconeogenesis” and “HIF-1 signaling pathway” as the two top processes upregulated under mild hypoxia (Figure 2C). In addition, the “Carbon metabolism” and “Central carbon metabolism in cancer” pathways were elevated, which have been shown to closely associate with the Warburg effect in cancer cells under the influence of hypoxia [33,34]. Interestingly, the “Notch signaling pathway” was also upregulated in neurons cultured at 5% O_2_. Although this finding from neurons was somewhat surprising, several earlier studies have established a crosstalk between hypoxia and Notch signaling, in which Notch-responsive promoters are activated by HIF1α, mediating expression of Notch-dependent downstream genes [35,36]. Meanwhile, neurons significantly downregulated 850 genes under mild hypoxic conditions (Figure 2B and Figure S1). Among these, the “Axon guidance” pathway was most affected, an intriguing finding consistent with reports that during brain development, hypoxia disrupts axon pathfinding and neuronal migration in various organisms, including *C. elegans*, zebrafish, and mammals [37,38]. Not surprisingly, hypoxia suppressed neuronal “Oxidative phosphorylation”, a process by which cells use oxygen to produce adenosine triphosphate (ATP) (Figure 2C).

Assessment of neuronal transcriptomes under 1% vs. 5% O_2_ revealed 1219 upregulated DEGs (Figure 2D and Figure S1). It is evident that further oxygen decline enhanced both the “HIF-1 signaling pathway” and “Glycolysis/Gluconeogenesis”, two pathways among the top three processes most represented in the significantly upregulated genes (Figure 2E). In addition, the “Circadian rhythm” pathway was affected in neurons cultured under 1% O_2,_ a finding in line with reports of the crosstalk between hypoxia and the circadian clock at the genome level [39,40]. Between 1% and 5% O_2_, neurons downregulated 1196 DEGs and profoundly repressed the “Cell cycle” pathway (Figure 2D,E and Figure S1). Hypoxia is known to alter cell cycle progression and interrupt various phases of cell proliferation [41,42]. Since neurons are post-mitotic, the immediate functional implication of this finding is unclear.

To comprehend the consensus molecular changes accompanying oxygen decline in neurons, we examined the shared DEGs between the neuronal transcriptomes at 5% vs. 21% O_2_ and those between 1% vs. 5% O_2._ We identified 343 genes that were consistently upregulated and 102 genes downregulated when neurons were confronted with continuous oxygen deprivation (Figure 2F,G) (Table S1). Remarkably, the genes with persistently increased expression belonged to the well-characterized hypoxia response pathways, such as the “HIF-1 signaling pathway”, “Glycolysis/Gluconeogenesis”, and “Central carbon metabolism in cancer”, revealing a highly conserved canonical hypoxia response in neurons under hypoxic conditions. No significant consensus pathway was identified from the shared downregulated gene list.

As hypoxia can rapidly reprogram chromatin [43], we next examined the expression of key epigenetic regulators in neurons during oxygen decline. The TET (ten-eleven translocation) proteins are demethylases that dynamically control DNA methylation patterns in various tissues. In response to hypoxia, neurons upregulated the transcription of *Tet1*, *Tet2*, and *Tet3* in a largely dose-dependent manner (Figure S2). The sirtuin family of proteins (SIRTs) are class III histone deacetylases that play complex and important roles in epigenetic modifications and chromatin regulation under diverse biological processes. We detected higher *Sirt4* and *Sirt6* levels in hypoxia-exposed neurons (Figure S2). SATB2 protein facilitates chromatin structure organization and coordinates the activity of multiple genes involved in the development. REST is a potent epigenetic repressor of neuronal genes during embryonic development, which normally remains downregulated in terminally differentiated neurons [44]. Although unaffected by mild hypoxia, both *Sirt1* and *Satb2* levels were significantly reduced while *Rest* mRNA markedly increased in neurons cultured under 1% O_2_ (Figure S2).

Altogether, these data have revealed a highly sensitive transcriptomic response of primary neurons to oxygen decline, a process accompanied by profound epigenetic reprogramming.

### 3.3. Transcriptional Responses of Astrocytes to Reduced Oxygen Availability

**Figure 3:**
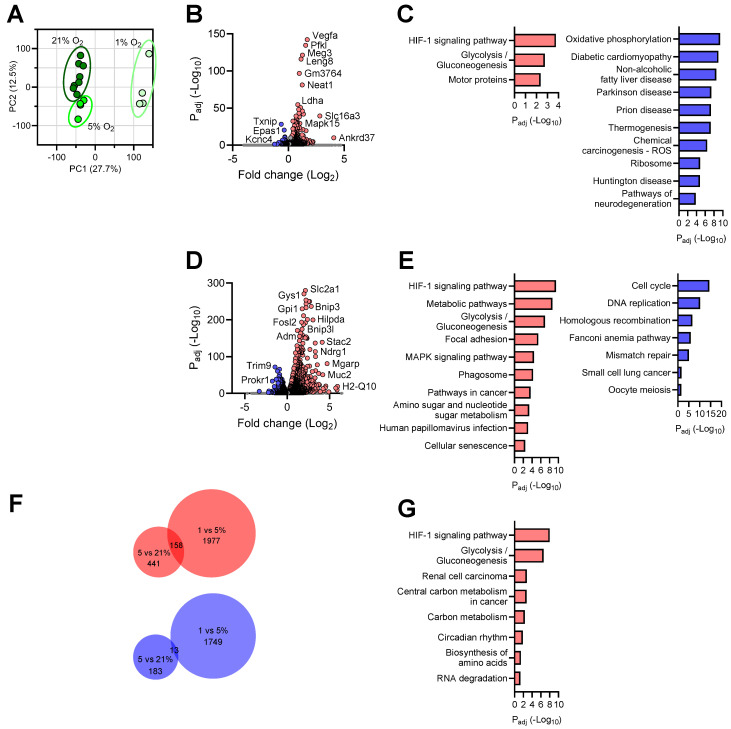
Transcriptional profile of astrocytes in response to different oxygen levels. (**A**): PCA plot of the transcriptomes of astrocyte cultures under different O_2_ conditions; (**B**): Volcano plot of significantly up- and downregulated genes at 5% vs. 21% O_2_ (FC > 1.2); (**C**): Top KEGG pathways enriched in up- (left) and downregulated (right) genes at 5% vs. 21% O_2_; (**D**): Volcano plot of significantly up- and downregulated genes at 1% vs. 5% O_2_ (FC > 1.2); (**E**): Top KEGG pathways enriched in up- (left) and downregulated (right) genes at 1% vs. 5% O_2_; (**F**): Venn diagrams showing the numbers of unique and shared DEGs (upregulated in red, downregulated in blue) between the two hypoxic conditions tested; (**G**): Top KEGG pathways enriched in upregulated genes shared between the two hypoxic conditions tested.

On the PCA plot, three treatment groups of astrocytes showed segregated clustering, indicating sizable transcriptomic differences among astrocytes under oxygen disparity (Figure 3A). Astrocytes exposed to mild hypoxia significantly upregulated the expression of 599 genes compared with normoxia (Figure 3B and Figure S1). Similar to neurons, the “HIF-1 signaling pathway” and “Glycolysis/Gluconeogenesis” constituted the top processes elicited by mild hypoxia treatment, indicating a characteristic hypoxic response (Figure 3C). Remarkably, the “Motor proteins” pathway was found elevated in astrocytes cultured under 5% O_2_. Corresponding to such process, multiple members of myosin, kinesin, and dynein family genes were significantly upregulated, an intriguing novel observation. Under mild hypoxic conditions, we found 196 genes that were significantly downregulated in astrocytes (Figure S1). As expected, the “Oxidative phosphorylation” pathway was found most significantly repressed (Figure 3C). Pathway analysis also identified other terms, such as “Diabetic cardiomyopathy” and “Non-alcoholic fatty liver disease” (Figure 3C), two peripheral disease conditions known to be critically modulated by hypoxia [45,46].

**Figure 4:**
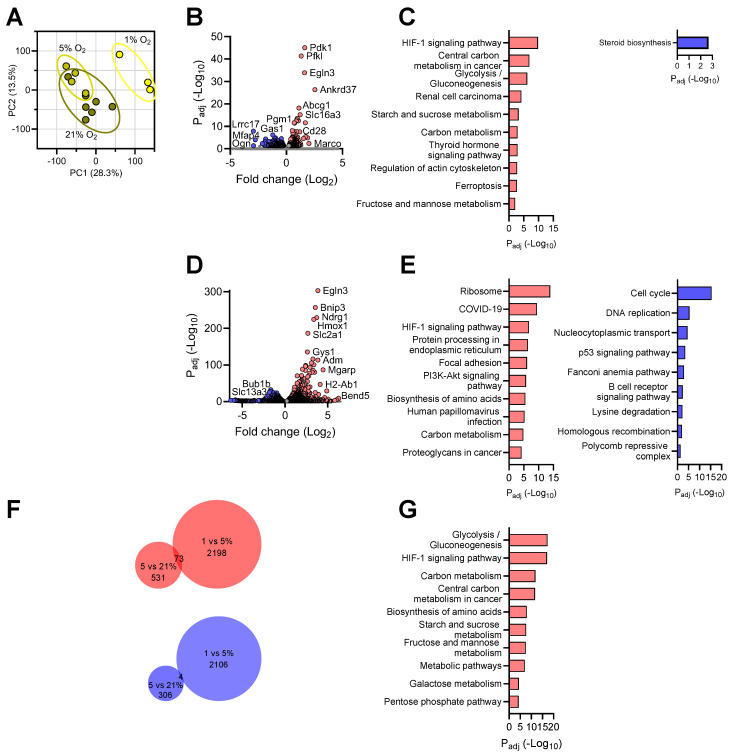
Transcriptional profile of microglia in response to different oxygen levels. (**A**): PCA plot of the transcriptomes of microglia cultures under different O_2_ conditions; (**B**): Volcano plot of significantly up- and downregulated genes at 5% vs. 21% O_2_ (FC > 1.2); (**C**): Top KEGG pathways enriched in up- (left) and downregulated (right) genes at 5% vs. 21% O_2_; (**D**)**:** Volcano plot of significantly up- and downregulated genes at 1% vs. 5% O_2_ (FC > 1.2); (**E**): Top KEGG pathways enriched in up- (left) and downregulated (right) genes at 1% vs. 5% O_2_; (**F**): Venn diagrams showing the numbers of unique and shared DEGs (upregulated in red, downregulated in blue) between the two hypoxic conditions tested; (**G**): Top KEGG pathways enriched in upregulated genes shared between the two hypoxic conditions tested.

Comparison of astrocytic transcriptomes at 1% vs. 5% O_2_ revealed 2135 upregulated DEGs, a more rigorous response than the one elicited by mild hypoxia (Figure 3D and Figure S1). Hypoxia-related pathways dominated the top processes affected by the DEGs, including “HIF-1 signaling pathway”, “Metabolic pathways”, and “Glycolysis/Gluconeogenesis” pathways (Figure 4E). Interestingly, the “Focal adhesion” pathway was also stimulated, a finding consistent with the observations that hypoxia activates and induces translocation of focal adhesion proteins to modulate the adhesive interaction between cells and the extracellular matrix [47]. When O_2_ levels reduced to 1%, the transcriptome of astrocytes contained 1762 genes with significantly lower expression in comparison with 5% (Figure 3E and Figure S1). Like neurons, the “Cell cycle” was significantly repressed. In contrast to neurons, the DEGs affected several pathways related to genomic DNA integrity, such as “DNA replication”, “Homologous recombination”, “Fanconi anemia pathway”, and “Mismatch repair”, indicating an astrocyte-specific response.

To probe the genes involved in persistent response by astrocytes to oxygen decline, we compared the DEGs identified above and identified 189 genes consistently affected by O_2_ reduction. Among these, 158 genes were steadily upregulated and 13 genes downregulated in the astrocytic transcriptomes with cumulative hypoxia exposure (Figure 3F,G) (Table S2). The “HIF-1 signaling pathway”, “Glycolysis/Gluconeogenesis”, “Central carbon metabolism in cancer“, and “Central carbon metabolism in cancer” constituted the foremost pathways associated with the upregulated DEGs, revealing a persistent canonical hypoxic response in brain astrocytes.

Therefore, we have detected significant transcriptomic responses by primary astrocytes to oxygen decline and identified biological pathways differentially affected by mild vs. severe hypoxia exposure.

### 3.4. Transcriptional Responses of Microglia to Reduced Oxygen Availability

Microglia cells cultured under normoxia and those with 5% O_2_ exhibited significant overlap on the PCA plot (Figure 4A), a feature distinct from neurons and astrocytes (Figure 2A and Figure 3A); yet, both clustered away from cells cultured under 1% O_2_ (Figure 4A), indicating that microglia responded more intensely to severe than mild hypoxia. By comparing the transcriptomes of microglia cultured under 5% O_2_ vs. those under normoxia, we detected 604 genes significantly upregulated under mild hypoxia condition (Figure 4B and Figure S1). These DEGs are primarily associated with hypoxia-related processes, such as the “HIF-1 signaling pathway”, “Central carbon metabolism in cancer”, “Glycolysis/Gluconeogenesis”, “Starch and sucrose metabolism”, and “Carbon metabolism” (Figure 4C). On the other hand, microglia significantly downregulated 379 genes under mild hypoxia (Figure S1). Pathway analysis of these genes identified “Steroid biosynthesis” as the sole significantly affected biological process (Figure 4C). Interestingly, neurosteroids can function as regulators of neuroinflammation, and several genes involved are also linked to lipid droplet formation, which integrates cell metabolism and innate immunity in myeloid cells [48,49].

A more drastic transcriptomic change was detected when we compared microglial transcriptomes at 1% vs. that at 5% O_2_: 2135 upregulated and 2110 downregulated transcripts were identified (Figure 4D and Figure S1). Unlike neurons and astrocytes, the most positively affected pathways in microglia under hypoxia are “Ribosome” and “COVID-19” in microglia (Figure 4E). Interestingly, several reports have revealed a significant impact of hypoxia on ribosomal function and protein synthesis in human cells [50,51], whereas numerous studies have established a close link between hypoxia and COVID-19 pathogenesis [8,52,53,54]. In addition, we detected an enhanced “HIF-1 signaling pathway”, and “Carbon metabolism”, indicating a prototypical hypoxia response in microglia exposed to lower oxygen levels (Figure 4E). Pathway analysis on the downregulated transcripts identified “Cell cycle”, “DNA replication”, “Fanconi anemia pathway” and “Homologous recombination” as the most significantly repressed processes in microglia under hypoxia (Figure 4E), analogous to the astrocytic response under the same condition (Figure 3E). Unique among brain cells, microglia depressed “Nucleocytoplasmic transport” and “p53 signaling pathway” when exposed to 1% O_2_ concentration.

We then compared the microglial DEGs identified above and identified 77 genes involved in continuous response by microglia to oxygen decline; among which, 73 genes were steadily upregulated and 4 genes downregulated in the microglial transcriptomes with cumulative hypoxia exposure (Figure 4F,G) (Table S3). Similar to other brain cells, the upregulated shared DEGs were primarily involved in a canonical hypoxia response that included “Glycolysis/Gluconeogenesis”, the “HIF-1 signaling pathway”, “Carbon metabolism”, and “Central carbon metabolism in cancer” pathways.

Hence, our transcriptomic analysis revealed that primary microglia are more tolerant to mild hypoxia than neurons in culture. While eliciting a canonical hypoxia response, the brain resident immune cells exhibit additional unique transcriptional responses to oxygen decline.

### 3.5. Configuration of the Hypoxia Response Machinery in Mouse Brain Cells

**Figure 5:**
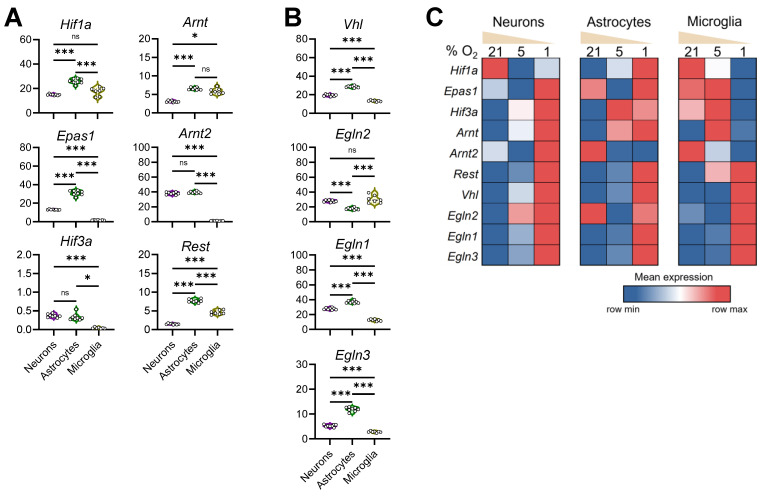
Distinct transcriptional profiles of hypoxia response machinery in brain cell types during normoxia and hypoxia. (**A**): Expression of members of the HIF family (*Hif1a*, Hif-2a encoded by *Epas1*, *Hif3a*, Hif-1b encoded by *Arnt*, and Hif-2b encoded by *Arnt2*), and *Rest* under normoxic conditions across cell types; ns: not significant (**B**): Expression of negative HIF regulators *Vhl*, and PHD1, -2, and -3 (encoded by *Egln2*, *Egln1*, and *Egln3*, respectively) under normoxic conditions across cell types; (**C**): Group-averaged relative expression of these transcripts under normoxia and reduced O_2_ levels. (***: *p* < 0.001, *: *p* < 0.05, ns: *p* > 0.05).

Given the observations of both shared and differential responses to decreasing oxygen, we then examined the core mediators of cellular hypoxia response in mouse primary brain cells. Under normoxia, astrocytes presented the highest levels of HIF-1α (encoded by *Hif1a*) and HIF-2α (encoded by *Epas1*) transcripts, two key O_2_-labile hypoxia-inducible factors, compared to neurons and microglia (Figure 5A). While the *Hif3a* transcript was extremely low in neurons and astrocytes, it was absent in microglia. Astrocytes also abundantly expressed HIF-1β (encoded by *Arnt*) and HIF-2β (encoded by *Arnt2*), subunits of HIFs known to be constitutively expressed. In comparison, primary neurons cultured under normoxia conditions maintained moderate expression of *Hif1a*, *Epas1*, and *Arnt* genes (Figure 5A). Microglia, on the other hand, expressed intermediate levels of HIF-1α and high levels of HIF-1β (*Arnt*)*,* but completely lacked the expression of HIF-2α (*Epas1*), HIF-3α, and HIF-2β (*Arnt2*) (Figure 5A). Hypoxia is known to stimulate upregulation as well as downregulation of gene expression. As mentioned above, REST has been known as a neuronal epigenetic regulator [44]. Recently, REST was identified as an important transcription repressor in hypoxic response [55,56]. Consistent with the literature, we observed highly repressed *Rest* expression in differentiated neurons. Interestingly, the highest expression of *Rest* was detected in primary astrocytes, and moderate levels of *Rest* transcript were found in microglia under normoxia (Figure 5A).

HIF alpha subunits are post-translationally regulated by VHL and PHD proteins during normoxia and stabilized under hypoxia conditions. We found that, under normoxia, primary astrocytes expressed the highest levels of *Vhl*, PHD2 (encoded by *Egln1*), and PHD3 (encoded by *Egln3*) and lowest levels of PHD1 (encoded by *Egln2*), when compared with neurons and microglia (Figure 5B). Microglia, on the other hand, expressed the lowest levels of *Vhl*, *Egln1*, and *Egln3*. Primary neurons positively and moderately express *Vhl*, *Egln2*, *Egln1*, and *Egln3* under normoxia. These findings revealed a distinct configuration of hypoxia response machinery in major brain cells.

To comprehend the transcriptional regulation of these hypoxia mediators, we compared the relative levels of these genes in each cell type when exposed to different O_2_ concentrations (Figure 5C). Remarkably, neuronal and microglial *Hif1a* levels were reduced, whereas astrocytic *Hif1a* increased, in response to mild hypoxia. When cultured at 1% O_2_, astrocytes elevated *Hif1a* transcription, while microglia repressed the expression of *Hif1a*. The expression pattern for HIF-2α (*Epas1*) also differed significantly among the brain cells—while neurons and astrocytes significantly upregulated its expression, microglia decreased *Epas1* transcription at 1% O_2_. While the absolute *Hif3a* levels remained very low in brain cells, they also showed fluctuating expression to oxygen deviation. Although HIFβ subunits are stably expressed under normoxia, we detected significant cell type-specific transcriptional alterations of both HIF-1β (*Arnt*) and HIF-2β (*Arnt2*) under different O_2_ levels (Figure 5C). On the other hand, transcripts of *Rest*, *Vhl*, *Egln1*, and *Egln3* were found invariably increased in brain cells cultured at 1% O_2_ (Figure 5C), revealing a profound change in the molecular configurations of hypoxia response machinery in brain cells under hypoxia.

### 3.6. Shared Brain Cell Responses to Oxygen Decline

**Figure 6:**
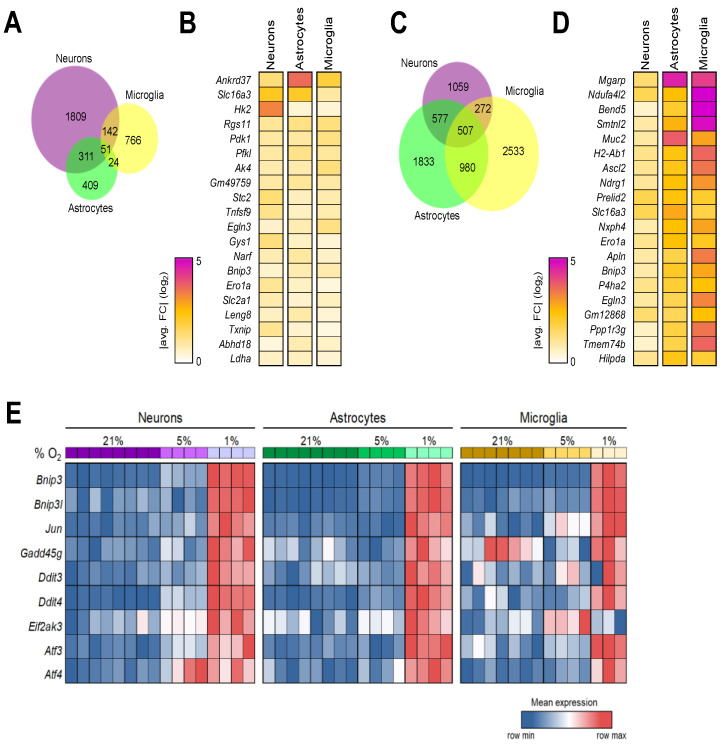
Shared transcriptional response to oxygen decline across brain cell types. (**A**): Venn diagram of overlapping DEGs between cell types at 5% vs. 21% O_2_; (**B**): Expression of top 20 DEGs across cell types by magnitude of fold change at 5% vs. 21% O_2_; (**C**): Venn diagram of overlapping DEGs between cell types at 1% vs. 5% O_2_; (**D**): Expression of top 20 DEGs across cell types by magnitude of fold change at 1% vs. 5% O_2_; (**E**): Relative expression of selected stress response genes across cell types at different oxygen levels.

We reasoned that, under brain hypoxia, all brain cells simultaneously undergo molecular reprogramming in response. Thus, it would be possible and worthwhile to establish a gene module to denote the brain-specific hypoxic response. For that, we analyzed the DEGs obtained from three brain cell types cultured under 5% O_2_ vs. those under normoxia and obtained 51 genes with significantly altered expression in all cells (Figure 6A) (Table S4). Among the top 20 genes with the most significant fold changes (Figure 6B), Ankyrin repeat domain 37 (*Ankrd37*) was previously identified as a HIF-1 target gene, and its human counterpart was recently linked to a novel causal mechanism controlling human hippocampal volume [57,58]. Similarly, solute carrier family 16 (*Slc16a3*) encodes one of the monocarboxylic acid transporters, is induced under hypoxic conditions, and subsequently promotes glycolytic metabolism in cells [59,60]. Also included in the list are several well-known genes involved in hypoxia response—*Hk2* [61], *Pdk1* (Figure 1), *Pfkl* [62], Ak4 [63], Stc2 [64], *Ero1a* [65], *Gys1* [66], *Egln3* (Figure 5), *Bnip3* [67], *Ldha* (Figure 1), and *Slc2a1* [68].

Furthermore, we identified 507 differentially expressed genes conserved among three brain cell types when the transcriptomes obtained from 1% vs. 5% O_2_ culture conditions were contrasted (Figure 6C). This is a much longer list than the one marking the brain cells’ shared response to mild hypoxia (Figure 6A), consistent with the observed stronger responses by glia cells to more significant hypoxia (Figure 3 and Figure 4). Figure 6D contains the top 20 DEGs with the greatest fold changes. *Mgarp* encodes mitochondria localized glutamic acid-rich protein and was expressed more than 3, 25, and 21 times in neurons, astrocytes, and microglia at 1% O_2_ over their counterparts at 5% O_2_ culture, respectively. A protein induced by hypoxia in neurons, Mgarp, also called mitochondrial movement regulator [69], has been shown to functionally modulate neocortical development and motility of neocortical neurons [70]. However, its presence and role in glial populations have not been investigated. In addition, we spotted other genes known to be involved in hypoxia response in the top 20 gene list—*Ndufa4l2* [71], *Muc2* [72], *Slc16a3* (see above), *Bnip3* (see above), *P4ha2* [73], *Egln3* (Figure 5), *Apln* [74], *Ero1a* [65], *Ascl2* [75], *Prelid2* [76], *Nxph4* [77], *Tmem74b* [78], and *Ndrg1* [79]. Of note, 28 of the 51 DEGs shared by brain cells in response to mild hypoxia were also represented in the gene module affected by significant hypoxia (Table S4).

BCL2/adenovirus E1B interacting protein 3 (*Bnip3*) appeared on both top 20 shared DEG lists (Figure 6B,D). *Bnip3* is known to be associated with diverse processes such as apoptosis, redox homeostasis, and glucose uptake regulation [80,81]. Expression of *Bnip3* and its binding partner *Binp3l* can be induced by HIF activation upon hypoxia exposure [67]. Via regulating mitochondrial function and mitophagy, the Bnip3/Bnip3l complex was shown to mediate hypoxia-induced cell death [82,83,84]. Both *Bnip3* and *Binp3l* transcripts were significantly elevated in brain cells cultured under 1% O_2_ (Figure 6E). As a part of MAPK cascades, the c-Jun N-terminal kinase (JNK) signaling pathway is involved in a wide range of cellular processes, particularly in the context of stress response and apoptosis [85]. We observed that primary brain cells invariably upregulated *Jun* expression under more severe hypoxia.

The DNA damage-inducible transcript 2 (*Ddit2*), alternatively known as GADD45G, is a member of the growth arrest and DNA damage-inducible gene family. It functions as a stress-responsive gene that is induced by genotoxic stress and other cellular stress signals. The DNA damage-inducible transcript 3 (*Ddit3*), also known as C/EBP homologous protein (CHOP), is a stress-inducible gene and regulates various cellular processes, including apoptosis, redox homeostasis, and glucose metabolism. The DNA damage-inducible transcript 4 (*Ddit4*) gene is induced by various cellular stresses and acts as a negative regulator of the mTOR pathway. Remarkably, 1% O_2_ exposure substantially increased the levels of these 3 stress response mediators in every brain cell type assayed in this study (Figure 6E).

The *EIF2AK3* gene encodes the protein kinase RNA-like ER kinase (PERK), which plays a key role in the unfolded protein response (UPR) to endoplasmic reticulum (ER) stress. Both neurons and astrocytes significantly upregulated *EIF2AK3* gene expression when cultured at 1% O_2,_ whereas microglia produced a higher level of *EIF2AK3* when cultured at 5% O_2._ The activating transcription factor 3 (*Atf3*) and activating transcription factor 4 (*Atf4*) are important stress-responsive genes that serve as master regulators of the cellular response to stress in various cellular processes, including the regulation of gene expression, metabolism, immunity, and oncogenesis. In primary neurons, *Atf3* and *Atf4* transcripts started to increase under mild hypoxia and reached higher levels when hypoxia grew more severe. Brain glia cells boosted *Atf3* and *Atf4* transcription primarily in response to lower O_2_ tension (Figure 6E).

In summary, our analyses have established hypoxia-driven brain gene modules and revealed profound stress-related responses in all brain cells under hypoxia.

## 4. Discussion

In this study, we conducted a comprehensive and unbiased genome-wide survey on the transcriptomes of major mouse brain cells that have been exposed to varied oxygen levels corresponding to normoxia, and mild and severe hypoxia. We not only detected cell type-specific responses, but our analysis also revealed that neurons, astrocytes, and microglia upheld a canonical hypoxia response to oxygen decline, which encompasses the well-characterized pathways such as the “HIF-1 signaling pathway”, “Glycolysis/Gluconeogenesis”, and “Carbon metabolism”, further validating the Nobel Prize-winning discoveries led by Drs. Gregg Semenza, William Kaelin, and Peter Ratcliffe.

For the first time, we established hypoxia-driven brain gene modules by identifying the shared DEGs among major brain cell types experiencing hypoxia (Figure 6). Despite broad pathophysiological impacts, non-life-threatening brain hypoxia is not routinely examined nor diagnosed, let alone studied in detail at the molecular level. Lacking reliable means to identify tissue hypoxia is apparently one of the contributing factors. Many biological processes, like hypoxia, can evoke rigorous cellular reactions, which lead to transcriptomic reprogramming affecting the expression of hundreds, if not thousands, of genes. Taking advantage of this notion, gene signatures tied to underlying pathogenic pathways have been successfully used in the diagnosis and monitoring of various human diseases [86,87,88]. The hypoxia-driven brain gene modules described here contain not only factors well-characterized in hypoxia response from other body cells and tissues but also gene entities that are brain specific or bear novel links. For example, our analyses highlighted *Mgarp*’s role in brain hypoxia beyond neurons, as it topped our shared brain gene signature panel (Figure 6D). The regulator of G protein signaling 11 (*Rgs11*) was also found significantly upregulated in all brain cells under mild hypoxia (Figure 6B). *Rgs11* is expressed in rodent brains in a region-specific manner [89]; yet, it has never been linked to hypoxia beforehand. Likewise, it would be interesting to further investigate the functional connections between genes, such as *Leng8*, *Bend5*, *Smtnl2*, *H2-Ab1*, and *Gm12868*, to brain hypoxia in future studies.

One of our primary goals in this study was to comprehend cell type-specific responses to hypoxia. Among brain cells, neurons and astrocytes are derived from neural stem cells or radial glial cells, whereas microglia originate from early embryonic erythromyeloid progenitors in the extra-embryonic yolk sac. With discrete transcriptomes (Figure 1B), these cells fulfill distinct functions in the brain. When exposed to mild hypoxia, neurons differentially expressed 2312 genes, extensively more than in glial cells—795 from astrocytes and 983 from microglia (Figure S1). The mechanism underlying such a rigorous response is unclear, as *Hif1a* and *Hif2a* are transcribed moderately in neurons. However, the levels of these proteins are primarily maintained via post-translational regulation, which could be affected by lower transcript levels of the negative regulators *Vhl*, *Egln1*, and *Egln3* in neurons than astrocytes under normoxia (Figure 5). On the other hand, microglia apparently express hypoxia response components in unique combinations, a pattern somewhat dissimilar to neurons and astrocytes (Figure 5). Comparing the transcriptomes from cells at 1% vs. 5% O_2_, we detected 2415 DEGs in neurons, 3987 in astrocytes, and 4312 in microglia, a contrast to their responses to mild hypoxia (Figure S1). Pathway analysis reveals that both neurons and astrocytes upregulate the “Circadian rhythm” and “MAPK signaling pathway“, and astrocytes and microglia upregulate “Focal adhesion”. In a cell specific-manner, neurons upregulate the “Notch signaling pathway” while downregulating “Axon guidance”, astrocytes upregulate “Motor proteins” and “Phagosome”, and microglia upregulate “Ribosome” and “COVID-19”.

Changes in oxygen availability have had a profound impact on the evolution of life on Earth. Mammals are highly adaptable to mild hypoxic conditions, such as high-altitude plateaus and subterranean burrows [90]. In animals and humans, the body’s oxygen delivery system produces an oxygen cascade, which results in varied physiological oxygen tensions in different tissues and organs [91]. Of note, the oxygen concentration inside the normal brain parenchyma is reportedly 4.4% ± 0.3% [91]. Interestingly, intermittent mild hypoxia has potential benefits to brain functions, such as increased working capacity and resistance to brain injury; moreover, it alleviates memory impairment in Alzheimer’s disease mouse models and patients with mild cognitive impairment [92,93,94,95]. In our dataset, both neurons and astrocytes cultured at 5% O_2_ exhibit significantly reduced the “Oxidative phosphorylation” (OXPHOS) pathway when compared with cells cultured at 21% O_2_. While OXPHOS is critical for efficient energy metabolism, it also serves as a source of reactive oxygen species and free radicals that promote oxidative stress. Oxidative stress has been implicated in the pathogenesis of several neurodegenerative diseases, including Alzheimer’s disease, Parkinson’s disease, and amyotrophic lateral sclerosis (ALS) [96]. In line with this, we observed remarkably downregulated processes, such as “Parkinson disease”, “Prion disease”, “Huntington’s disease”, and “Pathways of neurodegeneration—multiple diseases”, in astrocytes cultured at 5% O_2_ when compared to the 21% O_2_ condition.

On the contrary, severe hypoxia could be pathogenic and detrimental to tissues, especially for the oxygen-demanding brain. From this transcriptomic analysis, we have detected a multitude of molecular changes in brain cells exposed to intense hypoxia, which includes rigorous upregulation of canonical hypoxia response pathways, such as the “HIF-1 signaling pathway”, “Glycolysis/Gluconeogenesis”, and “Central carbon metabolism in cancer” (Figure 2, Figure 3 and Figure 4), significant reconfiguration of the hypoxia response machinery (Figure 5), and instigation of several core stress responses (Figure 6). Despite such a strain, cell death-related pathways were not apparently triggered in brain cells cultured under at 1% O_2_, nor was increased *Casp3* or *Casp8* expression detected. Of note, while playing a role in promoting axonal injury and neurodegenerative processes [97], *Ddit3/CHOP* was shown previously to protect neurons against hypoxia-induced cell death [21]. Thus, brain cells experiencing hypoxic stress undergo complex cellular changes, findings that are in need of further elucidation. Since chronic hypoxia profoundly shapes the cellular outcomes, analysis of additional time points would help discern other pathways induced following the acute response.

## 5. Conclusions

In summary, this study has produced a valuable resource that elucidates the transcriptional alterations occurring in brain cells in response to oxygen deprivation. Furthermore, we have identified hypoxia-driven gene modules specific to the brain, which may serve as a diagnostic tool for molecularly assessing brain hypoxia under pathophysiological conditions. The insights obtained into the cell-specific changes induced by hypoxia may also facilitate future research aimed at comprehensively understanding how this important cellular response shapes the brain and beyond.

## Data Availability

The data presented in this study are openly available in GEO (https://www.ncbi.nlm.nih.gov/geo/; accessed on 26 January 2024) under accession number GSE262728.

## Supplementary Materials

The following supporting information can be downloaded at: https://www.mdpi.com/article/10.3390/brainsci14040341/s1, Figure S1. Number of up- or downregulated genes between 1% vs. 5% or 5% vs. 21% across cell types (*P*_adj_ < 0.05, FC > 1.2). Figure S2. Relative expression of genes involved in epigenetic regulation in neurons upon treatments. (***: *p* < 0.001, **: *p* < 0.01, *: *p* < 0.05). Table S1. Lists of shared DEGs in neurons. Table S2. Lists of shared DEGs in astrocytes. Table S3. Lists of shared DEGs in microglia. Table S4. Lists of DEGs shared by brain cells.
