# Supplementary material for: Transcriptional Responses of Different Brain Cell Types to Oxygen Decline
Source: Brain Sci. 2024 Mar 30;14(4):341. doi: 10.3390/brainsci14040341 (PMC11048388; doi:10.3390/brainsci14040341)

Figure S1: DEGs per cell type and O2 levels

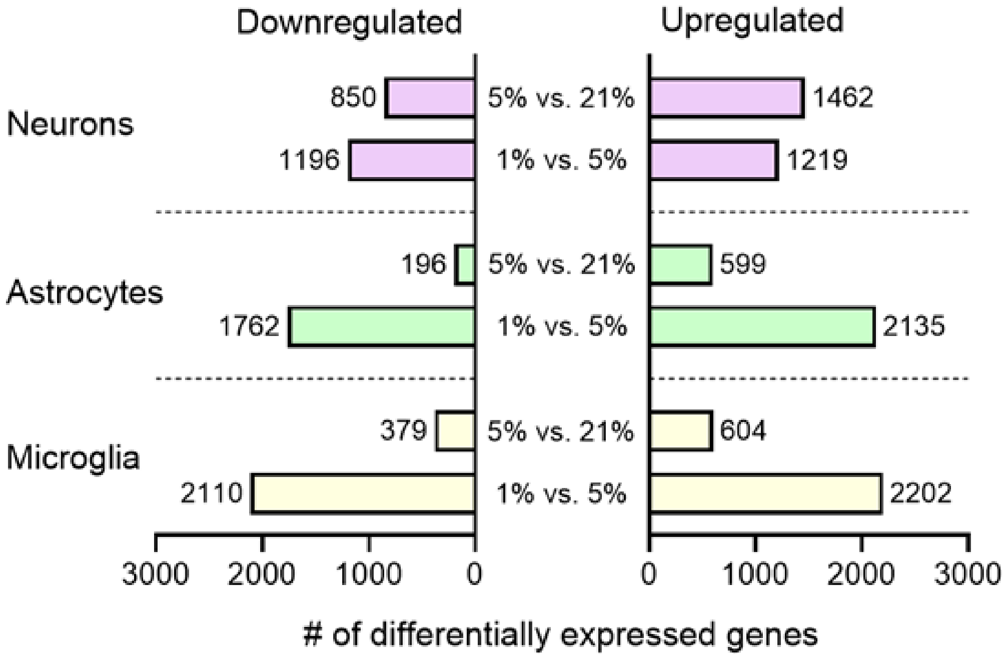

Fig. S2: Expression level of genes involved in epigenetic regulation in neurons

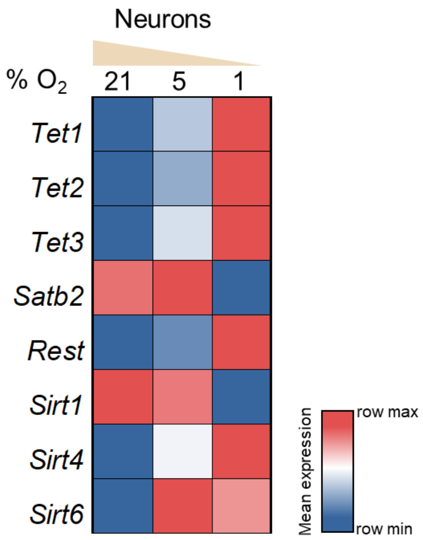

Supplement: Supplementary file 1 [file brainsci-14-00341-s001.zip › Hypoxia RNAseq suppl Figures.pdf]
